# From spawner habitat selection to stock‐recruitment: Implications for assessment

**DOI:** 10.1002/ece3.9679

**Published:** 2022-12-28

**Authors:** Stefan Skoglund, Rebecca Whitlock, Erik Petersson, Stefan Palm, Kjell Leonardsson

**Affiliations:** ^1^ Department of Aquatic Resources, Institute of Freshwater Research Swedish University of Agricultural Sciences Drottiningholm Sweden; ^2^ Department of Wildlife, Fish and Environmental Studies Swedish University of Agricultural Sciences Umeå Sweden

**Keywords:** behavior biology, fisheries management, habitat selection, population ecology, population productivity, stock‐recruitment

## Abstract

The relationship between the spawning stock size and subsequent number of recruits is a central concept in fisheries ecology. The influence of habitat selection of spawning individuals on the stock‐recruitment relationship is poorly known. Here we explore how each of four different spawner behaviors might influence the stock‐recruitment relationship and estimates of its parameters in the two most commonly used stock‐recruitment functions (Beverton‐Holt and Ricker). Using simulated stock‐recruitment data generated by four different spawner behaviors applied to multiple discrete habitats, we show that when spawners were distributed proportionally to local carrying capacities, there was small or no bias in estimated recruitment and stock‐recruitment parameters. For an ideal free distribution of spawners, larger bias in the estimates of recruitment and stock‐recruitment parameters was obtained, whereas a random and a stepwise spawner behavior introduced the largest bias. Using stock‐recruitment data corresponding to a “realistic” range of population densities and adding measurement error (20%–60%) to the simulated stock‐recruitment data generated larger variation in the estimation bias than what was introduced by the spawner behavior. Thus, for exploited stocks at low population density and where spawning stock size and recruitment cannot be observed perfectly, partial observation of the possible spawner abundance range and measurement error might be of higher concern for management.

## Graphical abstract

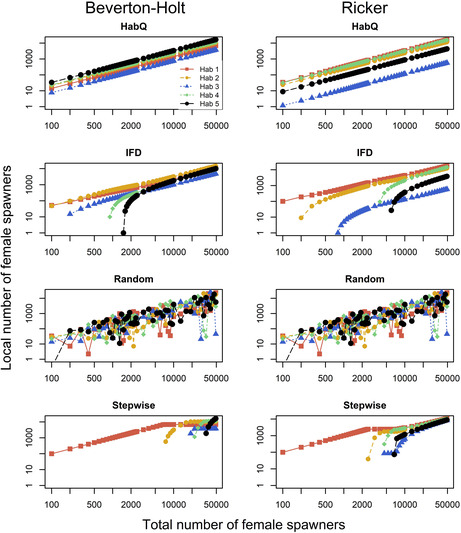

## 1 INTRODUCTION

Maximizing long‐term yield is a common target for the management of exploited fish populations around the world (Hilborn et al., 2015; Vert‐pre et al., 2013). The rate of harvest that maximizes yield strongly depends on a population's productivity (potential rate of net population increase), of which the maximum per capita recruitment at low population density (i.e. the slope at the origin of the stock‐recruit curve) is a key determinant (Beverton & Holt, 1957; Myers, 2001; Myers & Barrowman, 1996; Quinn & Deriso, 1999; Ricker, 1954). The productivity of an exploited population (stock), thus underlies its response to exploitation, and the level of harvest that it can sustain over the long term (Conn et al., 2010).

Stock‐recruitment (SR) models are widely applied in fisheries stock assessments to describe the expected average number of recruits as a function of spawning stock metrics (e.g. eggs or spawning stock biomass). The two most common SR models used in stock assessments are the Beverton‐Holt (Beverton & Holt, 1957) and the Ricker (Ricker, 1954) models. These two SR models have different functional forms, where recruitment increases asymptotically with increasing spawning output in a Beverton‐Holt model, whereas a Ricker model describes recruitment as a skewed dome‐shaped function of spawning output. Both models include parameters that are based on biological density‐dependent and ‐independent processes influencing the productivity of a population. As an example; the slope at the origin of the fitted curve in a SR model can be interpreted as the density‐independent maximum reproductive rate, whereas the asymptote (Beverton‐Holt model) or the maximum recruitment (Ricker model) describes density‐dependent population processes related to the system's carrying capacity (Myers, 2001). The shape of the SR relationship for a specific population correlates to both the evolved life history traits and the reproductive behavior (Foss‐Grant et al., 2016), where different stages in the pre‐recruited phase can be both density‐dependent and ‐independent (Brooks & Powers, 2007; Taylor et al., 2013).

Stock‐recruitment relationships form the basis for reference points (e.g. Maximum sustainable yield, MSY) that are commonly used to evaluate stock‐status and specify appropriate catch levels (Haddon, 2001). Thus, obtaining unbiased estimates of such parameters is crucial to avoiding loss of yield and/or unsustainable harvest rates in exploited natural populations (Needle, 2002). Ideally, estimated SR parameters should provide unbiased information on the productivity of a stock (Lee et al., 2012). However, fitting statistical functions to SRdata provides no insight into the biological mechanisms generating the observed patterns.

Estimated SR relationships are often uncertain and/or biased (Conn et al., 2010). A variety of factors influencing SR estimates has been suggested, e.g. time‐series bias (Walters, 1985), observation (Walters & Ludwig, 1981), and process errors (Linton & Bence, 2008), productivity regimes (Gilbert, 1997; Vert‐pre et al., 2013), and nonstationary dynamics (Feiner et al., 2015; Quinn & Deriso, 1999). Biological and ecological aspects such as age structure, spatial distribution, fecundity and spawning patterns are also known to influence the variation in recruitment (Green, 2008; Shelton et al., 2012). However, it remains poorly understood if (and how) the spatial distribution of spawning individuals may generate potential bias in SR estimates, although such behavioral patterns among spawners have long been acknowledged as a key factor affecting population productivity and regulation (Fretwell & Lucas, 1970; Jonzen et al., 2004; Morris, 1987; Pulliam & Danielson, 1991).

In fisheries applications, traditional SR models typically assume that the parameters determining productivity are constant across the range of reproductive output (Holt & Michielsens, 2020). For fish that spawn over large areas, this either that productivity is the same over the whole environment, or if it varies, that spawners distribute themselves in homogenously in the spawning environment. For many fish species, however, reproductive environments and the selection of habitats for reproduction among adults are known to deviate from these simplified assumptions (Bietz, 1981; Dingsør et al., 2007; Falcy, 2015; Finstad et al., 2013; Haugen et al., 2006; Purchase & Hutchings, 2008; Skjæraasen et al., 2011). Such deviations are expected to directly influence the initial intraspecific competition experienced by the offspring if the juvenile life‐stages are less mobile than the adults (as in many fish species). This may, in turn, ultimately influence population regulation if density dependence acts strongest in the initial life‐stages (Einum, Nislow, et al., 2008; Sinclair & Pech, 1996; Teichert et al., 2010; Turchin, 1999). As an example, for Atlantic salmon (*Salmo salar*), an anadromous species with heterogeneously distributed spawning aggregations in freshwater river habitats (Finstad et al., 2013; Fleming, 1996), it has been shown that juvenile survival is negatively correlated with egg density (Einum & Nislow, 2005). Thus, for some spawner behaviors, parameters determining population productivity and carrying might be influenced if the SR assumption of homogenous distribution of spawning individuals is violated.

In particular, the transfer or extrapolation of SR processes (i.e. maximum reproductive rates and carrying capacities) from one spatial scale to another can be problematic (Mayor et al., 2009), since patterns observed on one scale may not necessarily represent patterns at other scales (Levin, 1992). Data used for population‐scale SR estimates commonly represent a sum of many local nonlinear processes affected by multiple local productivity parameters. Available SR data typically consists of numbers or biomass of the spawning stock and resulting recruits; thus, different survival rates that may affect intermediate life stages (e.g. alevin and fry) are often unknown and subsumed into one estimated parameter. For example, density dependent regulatory processes act most strongly in the initial life‐stages on restricted temporal and spatial scales (Fernández‐Bellon et al., 2016; Finstad et al., 2009; Ray & Hastings, 1996; Rogers et al., 2017). A pre‐recruit multi‐stage model approach has been suggested to deal with the differences in density‐dependent and ‐independent survival in different life‐stages, which might improve the predicted number of recruits in a system (Brooks et al., 2019). Moreover, incorporating local rather than global density dependence in stock assessments is anticipated to provide a better understanding of the effects of fishing on spatially structured populations (Kapur et al., 2021). However, even if local SR parameters are known for all habitats in a system, SR estimates for the whole population might still be biased, if the distribution of spawners among those habitats is ignored. Density‐dependent processes might thus act under lower or higher than expected densities if spawner distributions deviate from the common SR assumption that productivity is not affected by the spatial distribution of spawners.

In this study, we study how population productivity might be affected by the underlying habitat selection patterns of spawning individuals. Moreover, we evaluate potential bias in estimates of recruitment and maximum survival rate over a range of population abundance, with and without realistic levels of measurement error, and what implication this might have in stock assessment context. Specifically, we have investigated if different spawner behaviors may introduce bias in estimates of recruitment as well as in SR parameter estimates, and how such bias might vary over population densities. This has been evaluated earlier, using the number of local spawning habitats as a proxy for the total carrying (Maunder & Deriso, 2013), but here we instead use local spawning area and local pre recruited survival rates as potential production capacity limitations. Using the widespread Ricker and Beverton‐Holt SR relationships applied to multiple discrete habitats, we compare simulated/expected and estimated total recruitment under four hypothetical spawner behaviors (*habitat quality*, *ideal free*, *random* and *stepwise*; definitions below). Following the spatial dispersal classifications from Thorson et al. (2016) and Bartolino et al. (2011), the habitat quality distribution would fit a constant density model, whereas the stepwise and ideal free distributions fit an proportional distribution model. The random distribution model does not fit any of the suggested distribution models, since distribution is not dependent on spawner abundance.

## 2 MATERIALS AND METHODS

Simulated data was produced to quantify potential bias in estimates of total recruitment (*R*′), total carrying capacity (*K*′) and maximum survival rate (*S*′) relative to underlying predetermined values, using two traditional SR‐functions (Beverton & Holt, 1957; Ricker, 1954). The SR‐functions used in this study do not account for density dependent mortality caused by predator behavior.

To disentangle the effects of the spawner behavior from other potential sources of bias (i.e. measurement error), and to achieve good contrast in the population abundance data, we first simulated data without additional measurement errors for a wide range of spawner abundances with a maximum close to carrying capacity. Second, to explore how potential effects of spawner behavior would manifest under a more realistic management scenario, we added measurement error and to a subset of the simulated recruitment data (only at lower spawner abundance levels). The simulations consisted of the following six steps:

1. Creation of different environments that define properties of local spawning sites, with randomly assigned habitat characteristics in terms of pre‐recruitment survival rates and carrying capacities.
2. Allocation of females to different spawning sites based on the four spawner behaviors evaluated and site‐specific environments.
3. Simulation of a recruits at each local site and under each spawner behavior, given the number of females present, using either the Beverton‐Holt or the Ricker functions.
4. Calculation of a predefined total carrying capacity and survival rate, using the local spawning site characteristics defined at step 1.
5. Estimation of the parameters for the two stock‐recruit functions and the total recruitment, using the total number of females over the entire population abundance range and the total recruitment.
6. Quantify the relative estimation bias (REB) as the difference between the estimated and the predefined: recruitment, survival rate and carrying capacity.

This procedure was then repeated for the measurement error and low abundance scenario using the same set of local parameter values. Each step is described in detail below.

### 2.1 Environments

In the equations and text that follow, subscript *i* denotes spawning site, *j* denotes the environment, and *k* denotes the spawner abundance level. In total, the simulations comprised 1000 different environments. Each spawning environment was assigned a unique set of local survival parameter, *S*1_*i*,*j*_ and carrying capacity *K*_*i*,*j*_, but was assumed to have the same female fecundity (*Fec*), array of total female numbers (*N*_*tot*_, range 100–50,000), and density‐independent egg‐juvenile survival, *S*0 (see Table 1 for definitions of parameters and variables). The stock‐recruit functions (Beverton‐Holt and Ricker) introduce density‐dependent mortality, which increases with an increasing number of spawners, and therefore the total survival rate will only reach *S*0 in situations with very few eggs.

**TABLE 1:** Definitions of terms, parameters and variables used in the simulations. The same parameter settings were used for all four spawner behaviors

Parameter	Values	Definition	Explanation
Environment	1–1000	Unique combinations of maximum survival rates and carrying capacity	The environment within which the population spawns. Each environment has five spawning sites
Spawning site	1–5	Five spawning sites per environment	Local spawning site. Each spawner selects one out of five available sites
*R*_*i*,*j*,*k*_	Varying, calculated	Local recruitment	Based on the underlying spawner behavior, the SR function and *N*_*tot*,*k*_
*R*_*tot*,*j*,*k*_	Varying, alculated	Total recruitment	The sum of recruitment from all spawning sites for the total spawner abundance
*N*_*i*,*j*,*k*_	Varying, predefined	Local female abundance	Local female abundance at each spawning site based on the spawner behavior for each *N*_*tot*,*k*_
*N*_*tot*,*k*_	Varying, predefined	Two arrays covering different total numbers of spawners per environment	Without measurement error: sequence of 49 abundances from 100–50,000. With measurement error: sequence of 24 abundances >1 but <*K*_*tot*,*j*_/2
*S*0	0.20	Same at all sites and for all environments	Density‐independent egg‐juvenile survival rate
*S*1_*i*,*j*_	0.05–0.30	Randomly drawn from a uniform distribution between 0.05–0.3 for each spawning site	Density‐independent survival rate covering the juvenile (including potential migration) phase
*K*_*i*,*j*_	0 < *K*_*i*,*j*_ < *K*_*tot*,*j*_	Randomly drawn from a Dirichlet multinomial distribution with a fixed total sum of local *K*_*i*,*j*_ (150,000 recruits) and with equal underlying probability parameter (*α* = 1) for all spawning sites	Local carrying capacity
*K*_*tot*,*j*_	150,000	Different depending on the SR function but the same for all environments	Total maximum carrying capacity in the environment, i.e. the sum of carrying capacities from the five local spawning sites
*Fec*	5000	Constant for all female spawners	Fecundity (number of eggs per female)
*logsd*_*m*,*j*_	0, 0.2, 0.4, 0.6	SD (of log(*x*)) for lognormal measurement errors	SD of lognormal measurement errors added to *R*_*tot*,*j*,*k*_ in the simulations with low spawner abundance levels
*χ*_*j*_	Varying, predefined	$\chi_{j}=\sum_{i=1}^{n}S1_{i,j}K_{i,j}$	Predefined maximum number of recruits for the environment *j*
ϴ_*j*,*k*_	Varying, predefined	${ϴ}_{j,k}=\frac{\sum_{i=1}^{n}R_{i,j,k}}{\sum_{i=1}^{n}\frac{R_{i,j,k}}{S1_{i,j}}}\;S0$	Predefined total survival rate for the environment *j* and spawner abundance *k*
*R*′_*j*,*k*_	Varying, estimated	Estimated total recruitment	Total recruitment estimated based on the number of recruits for spawner abundance *k* and environment *j*. Calculated from the SR functions using the estimates of *S*_*j*_′ and *K*_*j*_′
*K*_*j*_′	Varying, estimated	Estimated total carrying capacity	Estimated total (summed over all sites) carrying capacity in the environment *j*. Estimated from the entire range of simulated SR data
*S*_*j*_′	Varying, estimated	Estimated total survival rate	Estimated total survival rate in environment *j*. Estimated from the entire range of simulated SR data
*ω*_*tot*,*j*,*k*,*m*_	Varying, calculated	Total recruitment with measurement error	Total recruitment with additional measurement error with SD *m*

In each environment, there were five local spawning sites that the spawners could choose from (according to each evaluated spawning behavior). Each spawning site was associated with its own density‐independent survival *S*1_*i*,*j*_ and carrying capacity *K*_*i*,*j*_. *S*1_*i*,*j*_ covers the spawning site‐specific survival rate from juvenile to recruited individual leaving the spawning and nursery environment (e.g. river, seagrass meadow or bay). The sites were numbered from 1 to 5 with no. 1 having the highest density‐independent survival rate, *S*1_1,*j*_, followed by lower rates such that *S*1_5,*j*_ < *S*1_4,*j*_ < *S*1_3,*j*_ < *S*1_2,*j*_ < *S*1_1,*j*_. *K*_*i*,*j*_ parameters were randomly drawn from a Dirichlet‐multinomial distribution with a fixed total sum of *K i* = 1 to 5,*j* (150,000) for each environment and with the same underlying probability parameter (*α* = 1) for all spawning sites.

The environments were assumed constant over time, whereas the total number of females varied between years. It did not matter to the simulations in what order the total female numbers appeared; the actual years were not relevant to the results and were therefor omitted from the analyses. Hence, the dynamics of the populations are not modeled explicitly.

For each environment, two one‐dimensional arrays of female numbers were used. The first array contained 49 different total female numbers, with the highest numbers in a given environment chosen to reach close to *K*_*tot*,*j*_ in the Beverton‐Holt stock‐recruit function, and to exceed the peak in the Ricker function. The reason for this choice was to allow as good a fit as possible of the global SR function and to isolate the effects of spawner behavior, potentially causing bias in the recruitment estimates as well as in the parameter estimates. The second array consisted of 24 different equally spaced total female numbers, with the highest female numbers resulting in a total recruitment corresponding to just half of the total carrying capacity (*K*_*tot*,*j*_/2). This range of data availability was introduced in order to mimic real‐life management situations where harvested stocks often exhibit abundance levels far below carrying capacity.

Total recruitment (*R*_*tot*,*j*,*k*_) was calculated by summing local recruitment from the five spawning sites for each female abundance level after accounting for mortality loss due to *S*1_*i*,*j*_ and *S*0. Local recruitment (for each spawner behavior) was calculated using the Beverton‐Holt and the Ricker stock‐recruit functions. The Beverton‐Holt and Ricker functions were chosen since they are both widely used in stock assessments (Lowerre‐Barbieri et al., 2017; Myers, 2001; Walters & Martell, 2004), and moreover, since they cover two different compensatory shapes: asymptotic compensation (Beverton‐Holt) and dome‐shaped overcompensation (Ricker). The asymptotic compensatory process in the Beverton‐Holt function can arise from increasing intraspecific competition, whereas the over‐compensatory Ricker shape can be induced by cannibalism. There are other extensions of the Ricker and Beverton‐Holt SR functions that account for additional ecological theories (e.g. Maunder & Deriso, 2013; Taylor et al., 2013). The SR functions used in this study do not account for density dependent mortality caused by predator behavior.

We chose to parameterize both the Beverton‐Holt and Ricker functions using the peak level of recruitment (*K*_*i*,*j*_) (Pulkkinen & Mäntyniemi, 2013; Quinn & Deriso, 1999):

$$R_{i,j,k}=\frac{S1_{i,j}\;S0\;N_{i,j,k}\;\mathrm{Fec}}{1+S0\;N_{i,j},k\;\mathrm{Fec}/K_{i,j}},\;\left(\text{Beverton}-\text{Holt}\right)$$

$$R_{i,j,k}=S1_{i,j}\;S0\;N_{i,j,k}\;\mathrm{Fec}\;e^{-\frac{S0\;N_{i,j,k}\;\mathrm{Fec}}{e\;K_{i,j}}},\;\left(\text{Ricker}\right)$$

where $R_{i,j,k}$ is the local recruitment and $N_{i,j,k}$ is local female abundance. Carrying capacity, *K*_*i*,*j*_, can be thought of as the theoretical maximum recruitment the stock could obtain with maximum survival. Note that this differs from *R*0, another common parameterization defined as the long‐term average recruitment at demographic equilibrium with no fishing, as *R*_0_ also includes information about the unfished eggs or spawning biomass per recruit. The ratio of *R*_0_ to *K*_*i*,*j*_ depends on the stock‐recruit steepness: thus use of *K*_*i*,*j*_ is analogous to the use of *R*_0_ in situations where steepness approaches one. The relationship between *R*_0_ and *K*_*i*,*j*_ is described as:

$$R_{0,i,j}=\frac{K_{i,j}\left(\mathrm{EPR}0-\frac{1}{S1_{i,j}S0}\right)}{\mathrm{EPR}0},$$

where *EPR*0 is unfished eggs per recruit, which in our study would be *Fec*, since we assumed constant mean fecundity in all simulations.

#### 2.1.1 Spawner behaviors

The four spawner behaviors evaluated are defined as follows:

1. Preference for habitat quality, HabQ

The spawners' probability to select a local spawning site is directly proportional to the local habitat quality in terms of its carrying capacity compared with the other sites. In this scenario, the females will spread among the five local spawning sites such that the relative frequency distribution of spawners at the five sites becomes equal to the relative frequency distribution of the five carrying capacities. Note that this spawner behavior is not similar to what would be expected in a dynamic situation, at equilibrium, when the offspring returns to the site where they were born, as the HabQ behavior does not account for offspring migration mortality. The number of spawners in each local spawning site was calculated as:

$$N_{1,j,k}=\left(\frac{K_{1,j}}{\sum_{i=1}^{n}K_{i,j}}\right)N_{\mathrm{tot},k},N_{2,j,k}=\left(\frac{K_{2,j}}{\sum_{i=1}^{n}K_{i,j}}\right)N_{\mathrm{tot},k},\ldots N_{n,j,k}=\left(\frac{K_{n,j}}{\sum_{i=1}^{n}K_{i,j}}\right)N_{\mathrm{tot},k}$$

where *N*_*tot*,*k*_ is the total female abundance for the spawner abundance level *k*.

2 Ideal free distribution, IFD

Following an ideal free distribution, each spawner selects the spawning site that will maximize the overall per capita (Fretwell & Lucas, 1970). The first spawners will select the habitat with the highest product of *S*1_*i*,j_*K_*i*,*j*_, but as the number of spawners increase and density dependence starts to reduce the recruitment success the habitat choice will depend on the number of spawners already present at the different spawning sites. The solution in this situation can only be found by comparing the expected per capita recruitment success, $R_{i,j,k}/N_{i,j,k}$, at each spawning site. When there are many spawners the final distribution will be the one when the per capita recruitment is approximately the same at all spawning sites. At equilibrium the IFD will be identical to a strict homing spawner behavior (i.e. that spawners return to the same local site as where they were born), in terms of the number of spawners at each habitat, and in terms of individual fitness. The IFD process was solved iteratively so that each additional spawner “evaluated” the recruits/egg ratio based on the existing spawner densities at each spawning site.

3 Random habitat selection, Random

We used the Dirichlet‐multinomial distribution to distribute the spawners randomly among the five spawning sites:

$$N_{1,j,k},N_{2,j,k}\ldots N_{n,j,k}\sim \text{Dirichlet}-\text{Multinomial}\left(1,1,\ldots 1,N_{\mathrm{tot},k}\right).$$

With this function, the selection of spawning site is essentially random among the first spawners that arrive. With no influence from local habitat quality, whereas at an increased abundance spawners select sites in proportion to, approximately, twice the number of spawners already available at the sites. Hence, the distribution of spawners among sites starts to deviate from equal probabilities as the number of spawners increases. This means that the random distribution will seldom result in an equal distribution of spawners among the five sites. Moreover, the same distribution pattern is unlikely to be repeated in subsequent spawnings since the site that attracts many spawners is a random process.

4 Stepwise habitat selection, Stepwise

The stepwise habitat selection pattern was based on the theory of social attraction (Bietz, 1981), and the empirical study by Finstad et al. (2013), who showed that Atlantic salmon (Salmo salar) preferred areas used by other spawners, and that utilization of additional spawning sites was positively correlated with spawner densities. Under a stepwise habitat selection pattern, the closest (e.g. farthest downstream) spawning site (no. 1) will be used first until a fixed abundance threshold is reached, after which the next closest spawning site (no. 2) will be utilized, and so on until all habitats are filled. Then additional spawners are distributed equally among all spawning areas. The abundance threshold was set to 95% of *K*_*i*,*j*_, and equal for all spawning sites.

**FIGURE 1:**
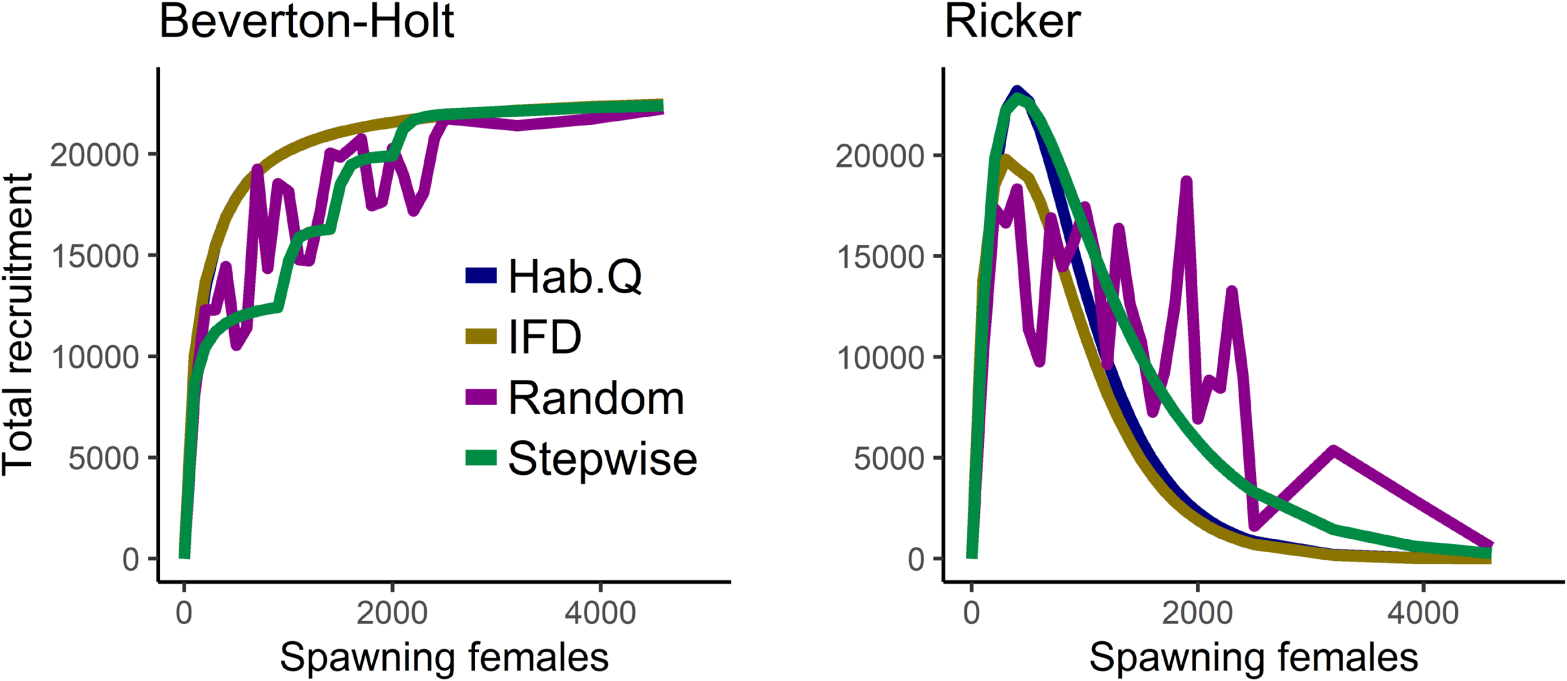
Conceptual figure showing differences in stock‐recruitment relationships for four different spawner distribution behaviors, assuming local Beverton‐Holt (left) and Ricker SR‐relationships (right). Spawners (females) and recruits have been summed across five local spawning sites. See text for details.

**FIGURE 2:**
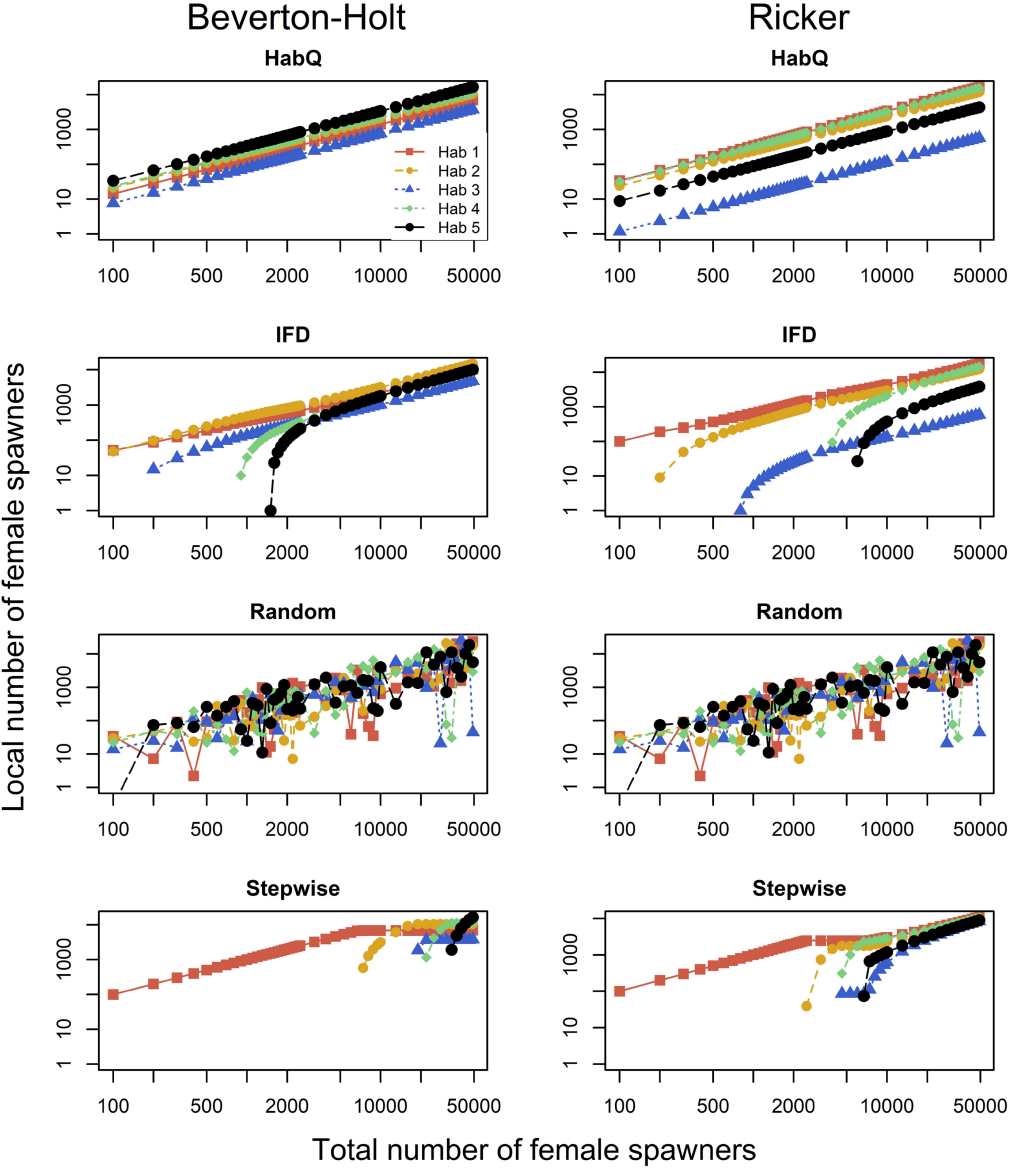
Total female abundance compared with the number of females at five local spawning sites with a common environment (environment 1). Panels represents combinations of the four spawner behaviors (rows) and two SR relationship (columns). Note that the two axes are shown on logarithmic scales.

The influence of the different spawner behaviors on the distribution of females among the five sites and on the total recruitment is illustrated in Figures 1, 2.

### 2.2 Parameter values used in the simulations

Values and limits for the predefined parameters were chosen arbitrarily, but are based on production parameters seen as realistic for a salmonid fish (according to expert opinion), Definitions and parameter values are listed in Table 1.

#### 2.2.1 Predefined parameter values

The predefined simulated parameter value for the maximum total recruitment for environment *j*, *χ*_*j*_, was defined as:

$$\chi_{j}=\sum_{i=1}^{n}S1_{i,j}\;K_{i,j}.$$

Predefined total survival rate in the system (ϴ_*j*,*k*_) was based on the five local survival rates (*S*1_*i*,*j*_) and the initial survival rate (*S*0) for each spawner abundance level, and was calculated as the product of total *S*1_*j*_ and S0 as:

$${ϴ}_{j,k}=\frac{\sum_{i=1}^{n}R_{i,j,k}}{\sum_{i=1}^{n}\frac{R_{i,j,k}}{S1_{i,j}}}S0,$$

where total *S*1_*i*,*j*_ is defined as the ratio of the total number of recruits based on the total *S*1_*i*,*j*_ and *S*0 ($\sum_{i=1}^{n}R_{i,j,k}$) and the total number of recruits with only *S*0 ($\sum_{i=1}^{n}R_{i,j,k}/S1_{i,j}$).

#### 2.2.2 Parameter estimation

For each of the four modeled spawner behaviors, the parameters *K*_*j*_′ (maximum recruitment in the environment) and *S*_*j*_′ (maximum survival rate in the environment) were estimated using the complete spawner abundance sequence (*N*_*tot*,*j*_, *n* = 49 or 24).

We estimated combined *S*_*j*_′ survival rate and carrying capacity, *K*_*j*_′, for the Beverton‐Holt model as:

$$R_{\mathrm{tot},j,k}=\frac{S_{j}\prime N_{\mathrm{tot},j,k}\mathrm{Fec}}{\left(1+{S_{j}}^{\prime }N_{\mathrm{tot},j,k}\mathrm{Fec}/{K_{j}}^{\prime }\right)},$$

and for the Ricker model as:

$$R_{\mathrm{tot},j}={S_{j}}^{\prime }N_{\mathrm{tot},j,k}\mathrm{Fec}\;e^{-\frac{S_{j}\prime N_{\mathrm{tot},j,k}\mathrm{Fec}}{e{K_{j}}^{\prime }}}.$$

Note that only one survival parameter was estimated, to resemble realistic stock assessment use of the SR functions where most often one density‐independent survival parameter (alpha) is estimated. *S*_*j*_′ here represented the combined survival rate from egg to recruit (i.e. *S*0 and *S*1_*i*,*j*_) as with ϴ_*j*,*k*_. Note also that the predefined survival rates (ϴ_*j*,*k*_) are defined for each spawner abundance level (that can imply a different distribution of spawners across sites), while the estimated survival rate does not depend on the spawner abundance. The parameters were estimated via non‐linear least squares regression using the “nls_multstart” function from the nls.multstart package in R (Padfield & Matheson, 2018). This function requires upper and lower starting values for the parameters estimated; upper and lower values were set to 0.001 and 0.1 for the *S*_*j*_′ parameter and to 10,000 and 40,000 for the *K*_*j*_′ parameter. These limits for the *K*_*j*_′ parameter were chosen since the maximum total recruitment in an environment would be a function of *S*1_*i*, *j*_ (Table 1). Thus, the maximum total recruitment in the environment would not be the sum of the total *K*_*i*,*j*_ (150,000).

#### 2.2.3 Relative estimation bias

The relative estimation bias (REB) was calculated as the difference between the estimated parameter values for *K*_*j*_′ and *S*_*j*_′, calculated from the SR data for the entire spawner abundance range, and their corresponding predefined values (*χ*_*j*_ and ϴ_*j*,*k*_), divided by the predefined parameter values, where positive and negative values indicate over‐ and under‐estimation, respectively. REB for *R*_*j*_′ was calculated as the difference in percent between the estimated recruitment (based on the SR curves obtained when applying the estimated *S*_*j*_′ and *K*_*j*_′ in the SR functions), and the observed recruitment from the simulations for the total range of spawner abundance. The SR data, analysis, parameter estimates and figures were executed and produced in R version 4.2.1 (R Core Team, 2022).

### 2.3 Measurement error and data availability for management situations

Based on the same 1000 environments 24 equally spaced spawner abundance levels were produced from a spawner abundance between 1 and *K*_*tot*,*j*_/2 where additional lognormal measurement error was added to the total recruitment for each spawner behavior, spawner abundance level and the two SR functions. In this simulation, the predefined parameter values for each environment were the same as above, and the REB calculations were made using the same predefined values of recruitment *R*_*tot*,*j*,*k*_, ϴ_*j*,*k*_ and *χ*_*j*_. Since a Ricker SR curve has two levels of spawner abundance representing *K*_*tot*,*j*_/2, the upper limit was set to *K*_*tot*,*j*_ and below in order to exclude levels of spawner abundances above the peak. To mimic realistic levels of measurement error, lognormal random variates with an SD of 0, 0.2, 0.4 or 0.6 were added to each observed total recruitment for each environment:

$$\omega_{\mathrm{tot},j,k,m}=\text{Lognormal}\left(\text{mean}=\log\left(R_{\mathrm{tot},j,k}\right),\sigma ={\text{logsd}}_{m}\right),$$

where *logsd*_*m*_ is the standard deviation of the lognormal distribution (SD of log(*x*)), and *m* indexes different magnitudes of measurement error (SD = 0, 0.2, 0.4 or 0.6), where SD = 0 depicted estimates without measurement error. Measurement errors typically depend on the type of observation/data and the process by which they are sampled or measured, and have been suggested to be around 60% among several orders of marine fishes (Thorson et al., 2014). However, a range between 0% and 20% measurement error is often sufficient to explore the consequences of both small and large measurement error in fisheries ecology (Memarzadeh et al., 2019), which is why we also included intermediate levels of measurement errors. Walters and Ludwig (1981) showed that lognormal measurement errors introduce bias in the stock‐recruit estimates as well as in the parameter estimates, and that this bias could be removed by multiplying with a correction term, exp(−1/2 *σ* ^2^). In this study, we applied this correction factor in order to analyze the bias introduced by the behaviors rather than that introduced by the lognormal measurement error.

## 3 RESULTS

The total recruitment and the SR parameters were affected by the spawning behavior, where the magnitude and variation in the REB dependent on the specific spawner behavior.

**TABLE 2:** Mean and SD of the median REB for the estimated *R*′, *S*′ and *K*′ based on additional measurement errors at different levels (SD) under Beverton‐Holt and Ricker SR relationships

Spawner behavior	Measurement error (SD)	*R´*		*S´*		*K´*	
		Mean	SD	Mean	SD	Mean	SD
Beverton‐Holt							
HabQ	0	0.00	0.00	0.00	0.00	0.00	0.00
	0.2	0.021	0.070	0.010	0.19	0.040	1.80
	0.4	0.087	0.17	0.10	0.51	0.090	434.25
	0.6	0.22	0.32	0.26	6.51	0.22	44747.62
IFD	0	−0.010	0.010	−0.060	0.070	−0.090	0.12
	0.2	0.020	0.070	−0.040	0.20	−0.11	1.12
	0.4	0.090	0.17	0.060	0.49	−0.090	127.36
	0.6	0.19	0.32	0.13	115.95	0.060	5231.92
Random	0	−0.03	0.29	−0.32	Inf	−0.17	0.22
	0.2	0.00	0.32	−0.29	0.44	−0.16	0.53
	0.4	0.07	0.38	−0.25	138.87	−0.090	4.91
	0.6	0.19	0.53	−0.18	2.41 × 10^7^	0.00	51.040
Stepwise	0	−0.01	0.26	−0.64	Inf	−0.41	Inf
	0.2	0.01	0.29	−0.64	0.85	−0.41	8865.13
	0.4	0.07	0.35	−0.63	467.88	−0.36	8962.090
	0.6	0.20	0.51	−0.60	8.53 × 10^6^	−0.27	56899.65
Ricker							
HabQ	0	0.00	4.12 × 10^−6^	2.11 × 10^−7^	3.56 × 10^−6^	−8.02 × 10^−7^	1.20 × 10^−5^
	0.2	0.030	0.070	0.040	0.14	−0.010	45.83
	0.4	0.11	0.18	0.12	0.30	0.12	631.36
	0.6	0.26	0.35	0.34	0.70	0.040	597.00
IFD	0	−0.010	0.010	−0.050	0.070	−0.28	0.31
	0.2	0.010	0.080	−0.030	0.15	−0.31	5.35
	0.4	0.080	0.18	0.050	0.29	−0.27	85.67
	0.6	0.21	0.33	0.19	0.57	−0.22	139.93
Random	0	−0.030	0.51	−0.20	0.32	−0.42	Inf
	0.2	0.010	0.55	−0.19	0.34	−0.38	2.37
	0.4	0.080	0.65	−0.13	0.41	−0.34	14.030
	0.6	0.21	0.89	−0.030	0.71	−0.25	57.040
Stepwise	0	−0.030	0.050	−0.23	0.27	−0.35	Inf
	0.2	0.00	0.09	−0.20	0.27	−0.32	18.38
	0.4	0.070	0.18	−0.15	0.31	−0.27	90.43
	0.6	0.22	0.37	0.010	0.51	−0.20	360.020

*Note*: Estimates only for lower ranges of spawner abundances (≤*K*_*tot*,*j*_/2). Inf (infinity) values were obtained when the exponential part of the SD calculation gave inf. Large numbers.

### 3.1 Relative estimation bias without measurement error and full range of spawner abundance

**FIGURE 3:**
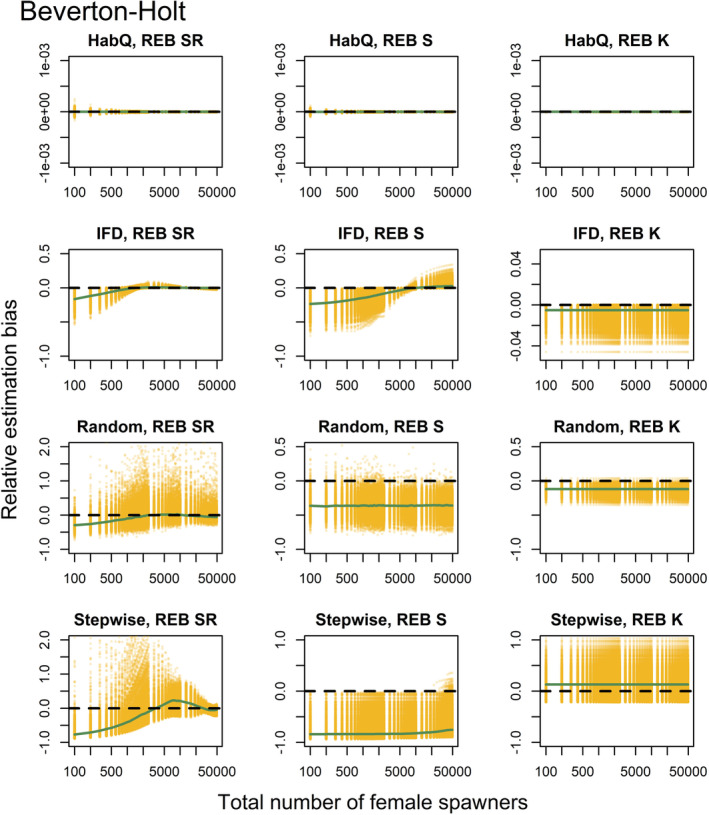
The REB for the total recruitment *R*′, survival rate *S*′ carrying capacity *K*′, for a Beverton‐Holt SR relationship. Each row represents one of the four different spawner behaviors evaluate (HabQ, IFD, random, stepwise). Each yellow dot represents REB calculated for a specific combination of an environment and a total spawner abundances. Green solid lines show the median REB calculated for each spawner abundance level from all of the 1000 environments. Solid black lines illustrate zero REB. Positive and negative values of REB indicate over‐/underestimation of the known parameter values and total recruitment. Note that the total number of female spawners (x‐axis) is shown using a logarithmic scale.

**FIGURE 4:**
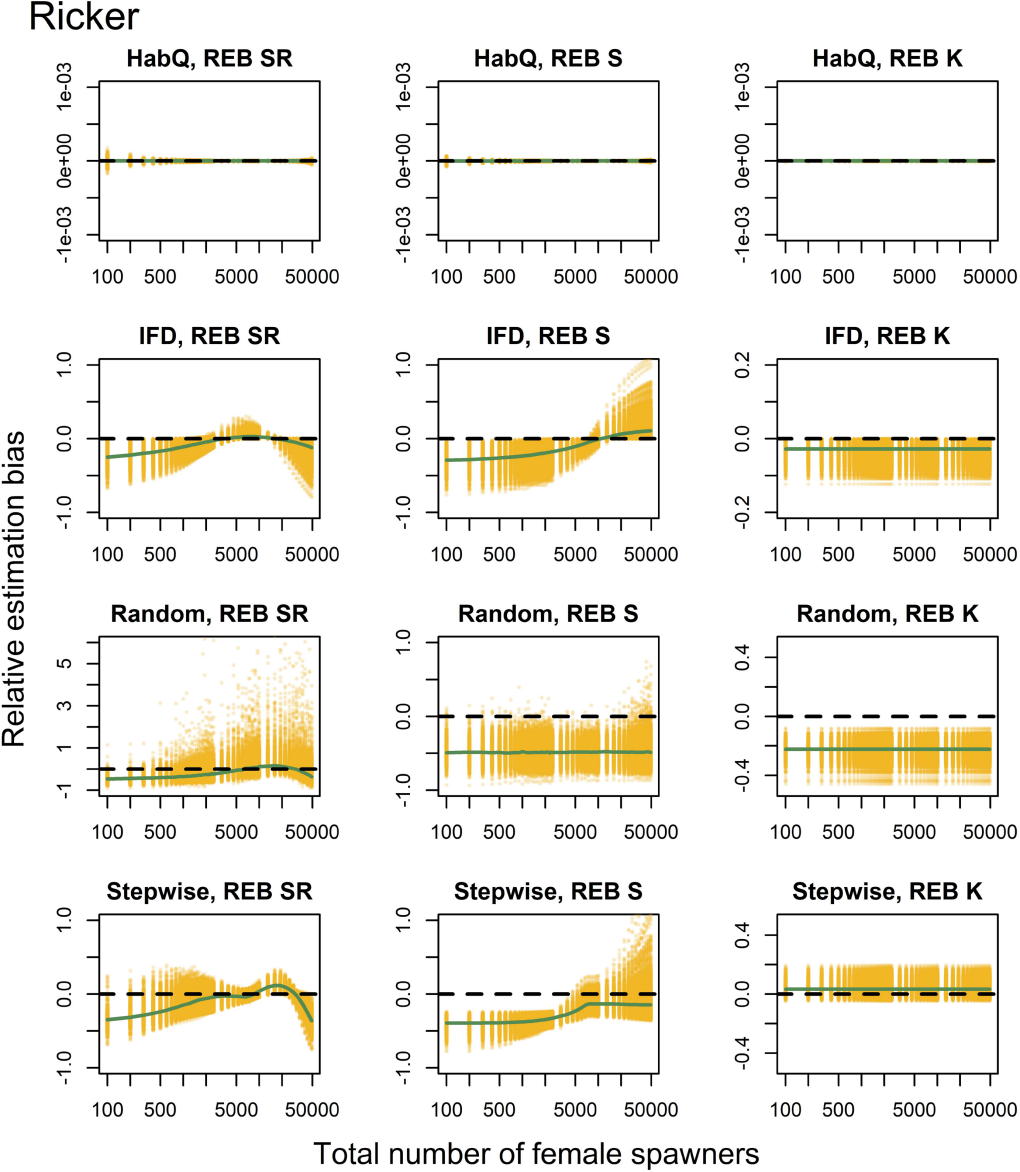
The REB for the total recruitment *R*′, survival rate *S*′ carrying capacity *K*′, for a Ricker SR relationship. Each row represents one of the four different spawner behaviors evaluate (HabQ, IFD, random, stepwise). Each yellow dot represents REB calculated for a specific combination of an environment and a total spawner abundances. Green solid lines show the median REB calculated for each spawner abundance level from all of the 1000 environments. Solid black lines illustrate zero REB. Positive and negative values of REB indicate over‐/underestimation of the known parameter values and total recruitment. Note that the total number of female spawners (x‐axis) is shown using a logarithmic scale.

In general, the estimates of recruitment (*R*_*j*_′) and the two parameters *S*_*j*_′ and *K*_*j*_′ were affected by the underlying spawner behavior, but the magnitude of observed bias (REB) was largely dependent on the distribution behavior (Figures 3, 4).

### 3.2 HabQ

Across environments, the HabQ spawner selection behavior did not introduce any bias in the prediction of total recruitment (*R*_*j*_′) or in the estimates of *S*_*j*_′ and *K*_*j*_′ (Figures 3, 4). This was consistent for both the Beverton‐Holt and the Ricker function (Figures 3, 4).

### 3.3 Ideal free distribution

With a Beverton‐Holt SR relationship, IFD spawner behavior caused larger bias in the estimated *R*_*j*_′, *S*_*j*_′ and *K*_*j*_′ compared with the HabQ spawner behavior (Figure 3). However, the REB in the *K*_*j*_′ parameter estimate was close to zero and constant over the full range of spawner abundance (Figure 3). Meanwhile, estimates of *R*_*j*_′ and *S*_*j*_′ introduced larger REB that was not constant over the spawner abundance range, indicating that with a Beverton‐Holt SR relationship the REB for these two estimates were dependent on the spawner abundance, where most REB was introduced at low spawner abundance levels (Figure 3).

Also with a Ricker SR relationship, the HabQ spawner behavior produced negligible REB, whereas with an IFD spawner behavior REB was almost twice as large on average compared with the Beverton‐Holt SR relationship (Figure 4). The REB in the *R*_*j*_′ and *S*_*j*_′ estimates varied over the spawner abundance range, whereas the REB introduced in *K*_*j*_′ estimates was constant (Figure 4). As for the Beverton‐Holt SR, a Ricker SR relationship introduced most REB for the *R*_*j*_′ and *S*_*j*_′ at low spawner levels, whereas the median REB at high spawner abundance was close to zero (Figure 4).

### 3.4 Random

The random spawner behavior introduced a large REB with significant variation compared with the other three spawner behaviors (Figures 3, 4). This was consistent for both the Beverton‐Holt and the Ricker SR functions. The estimates of *R*_*j*_′ were in general dependent on the spawner abundance, whereas the REB in the *K*_*j*_′ and *S*_*j*_′ was constant and independent of spawner abundance (Figures 3, 4). For the estimates of the parameters *K*_*j*_′ and *S*_*j*_′ virtually all of the REB was below zero, which means that in almost all environments these two parameters would be underestimated for a random type of spawner behavior (Figures 3, 4).

### 3.5 Stepwise

For both SR relationships, the stepwise spawner behavior introduced REB in the estimates of *R*_*j*_′, following the same pattern as for the other three spawner behaviors, with larger REB at low spawner abundance and REB close to zero at high spawner abundance (Figures 3, 4). However, the REB in the estimates of *R*_*j*_′ with a Ricker SR relationship dropped off at high spawner abundance (Figure 4). This means that for population abundances above the peak of the Ricker curve we would underestimate total recruitment. The REB in the estimates of *S*_*j*_′ were virtually independent of spawner abundance (constant) for the Beverton‐Holt SR relationship (Figure 3), but dependent on spawner abundance in the Ricker SR relationship (Figure 4). The REB for the *K*_*j*_′ estimates were in general overestimated, for both a Beverton‐Holt and a Ricker SR relationship (Figures 3, 4), but without any trends across the full range of spawner abundance.

### 3.6 Relative estimation bias at low population abundance, with and without measurement error

**FIGURE 5:**
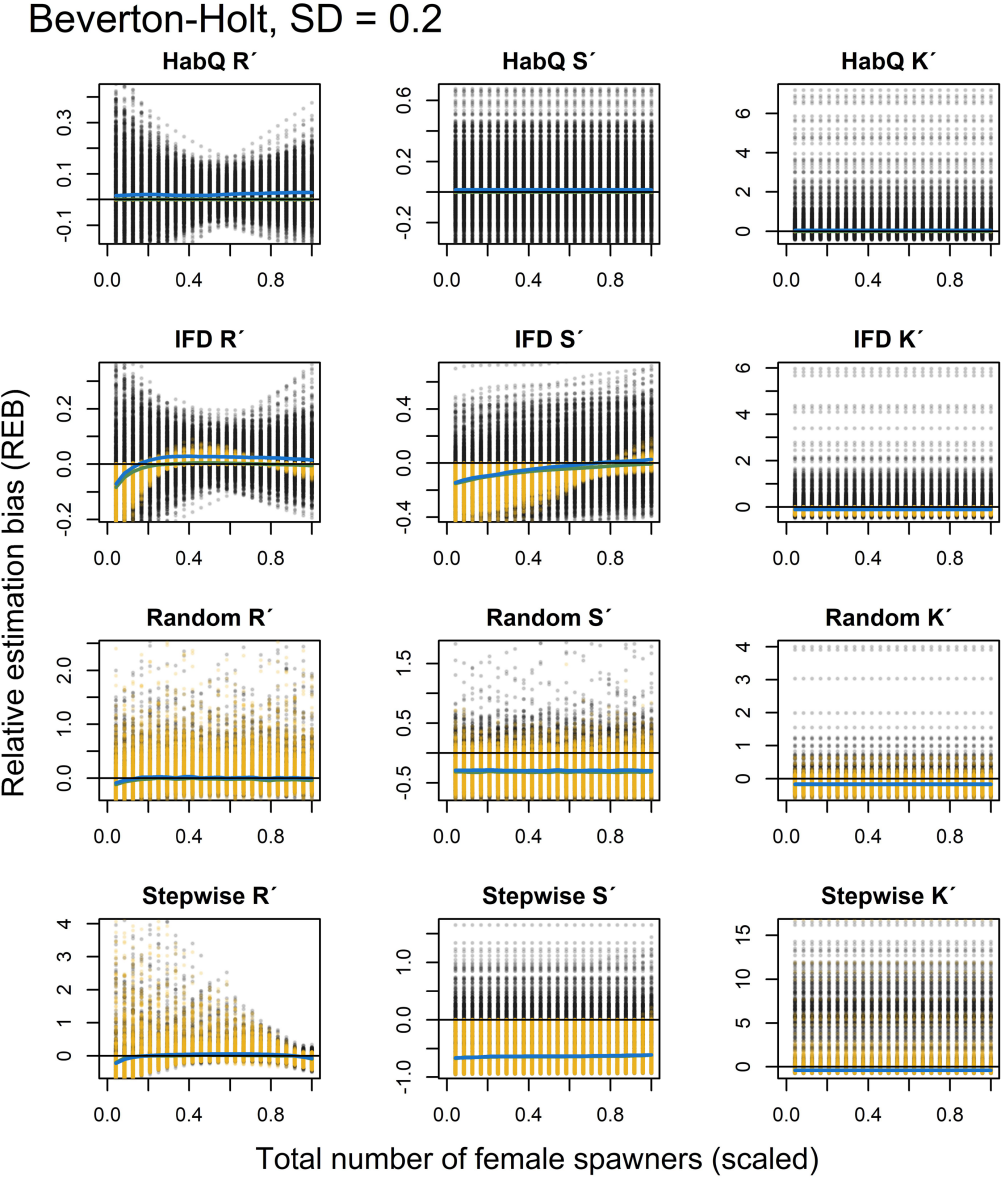
REB for *R*′, *S*′ and *K*′, based on low spawner abundance (*K*_*tot*,*j*_/2) without (yellow dots) and with added measurement error (SD = 0.2, black dots), for a Beverton‐Holt SR relationship. Green and blue solid lines show the median REB without and with measurement error, respectively. Black solid line shows zero REB. Note the different scales on the Y‐axis. For clearer visualization, female abundance (x‐axis) is displayed as a proportion of the evaluated maximum abundance.

**FIGURE 6:**
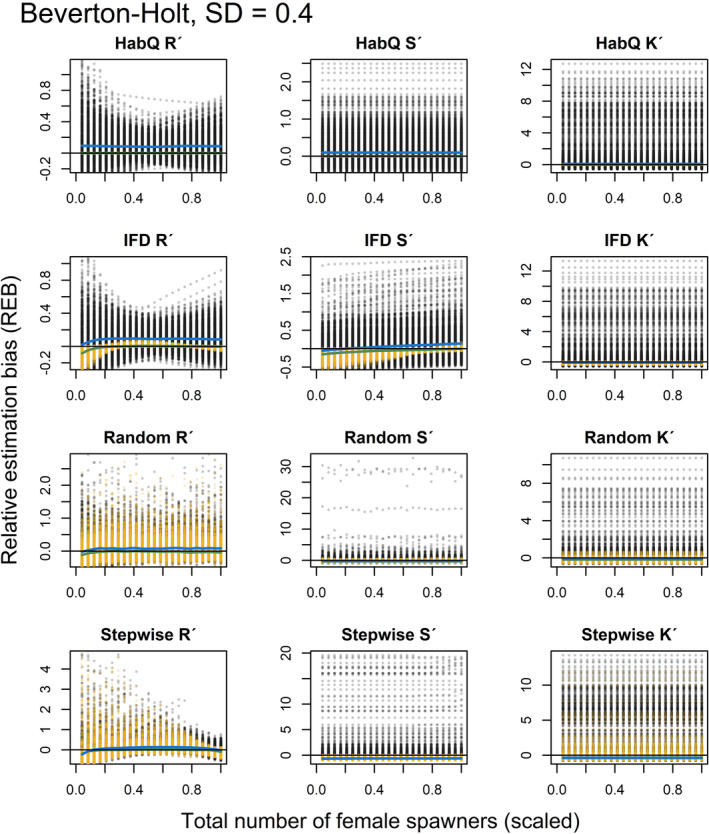
REB for *R*′, *S*′ and *K*′, based on low spawner abundance (*K*_*tot*,*j*_/2) without (yellow dots) and with added measurement error (SD = 0.4, black dots), for a Beverton‐Holt SR relationship. Green and blue solid lines show the median REB without and with measurement error, respectively. Black solid line shows zero REB. Note the different scales on the Y‐axis. For clearer visualization, female abundance (x‐axis) is displayed as a proportion of the evaluated maximum abundance.

**FIGURE 7:**
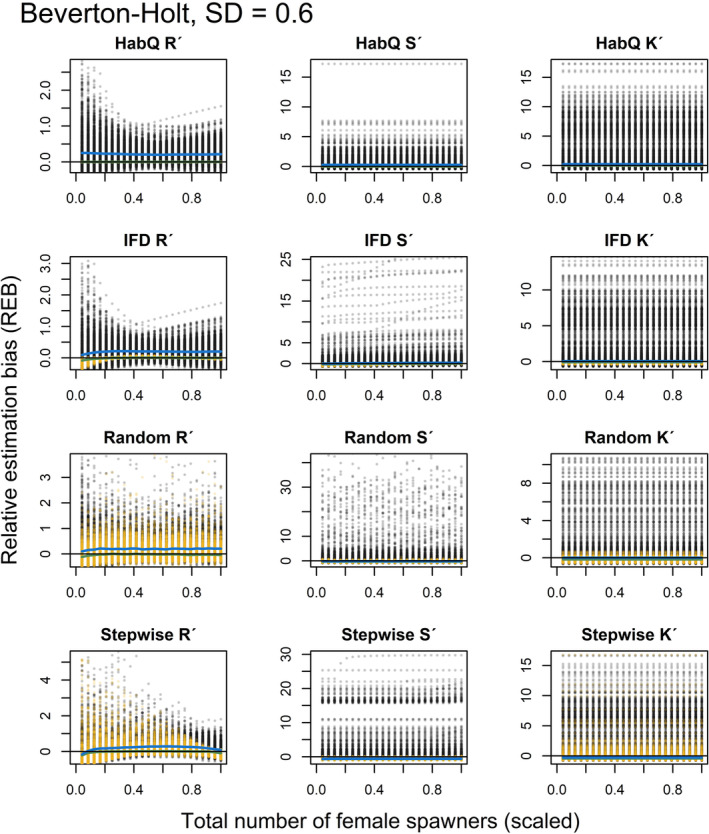
REB for *R*′, *S*′ and *K*′, based on low spawner abundance (*K*_*tot*,*j*_/2) without (yellow dots) and with added measurement error (SD = 0.6, black dots), for a Beverton‐Holt SR relationship. Green and blue solid lines show the median REB without and with measurement error, respectively. Black solid line shows zero REB. Note the different scales on the Y‐axis. For clearer visualization, female abundance (x‐axis) is displayed as a proportion of the evaluated maximum abundance.

**FIGURE 8:**
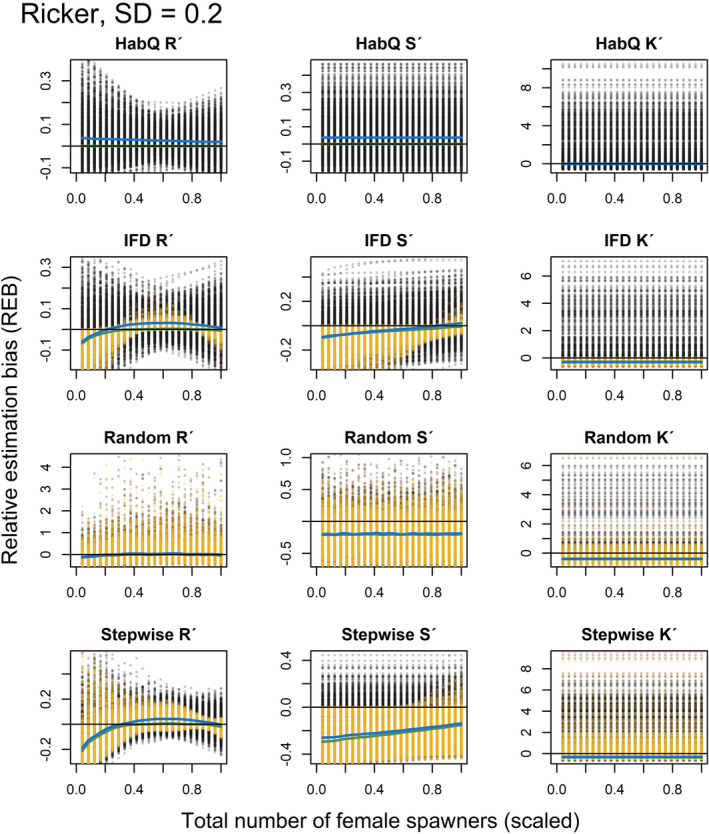
REB for *R*′, *S*′ and *K*′, based on low spawner abundance (*K*_*tot*,*j*_/2) without (yellow dots) and with added measurement error (SD = 0.2, black dots), for a Ricker SR relationship. Green and blue solid lines show the median REB without and with measurement error, respectively. Black solid line shows zero REB. Note the different scales on the Y‐axis. For clearer visualization, female abundance (x‐axis) is displayed as a proportion of the evaluated maximum abundance.

**FIGURE 9:**
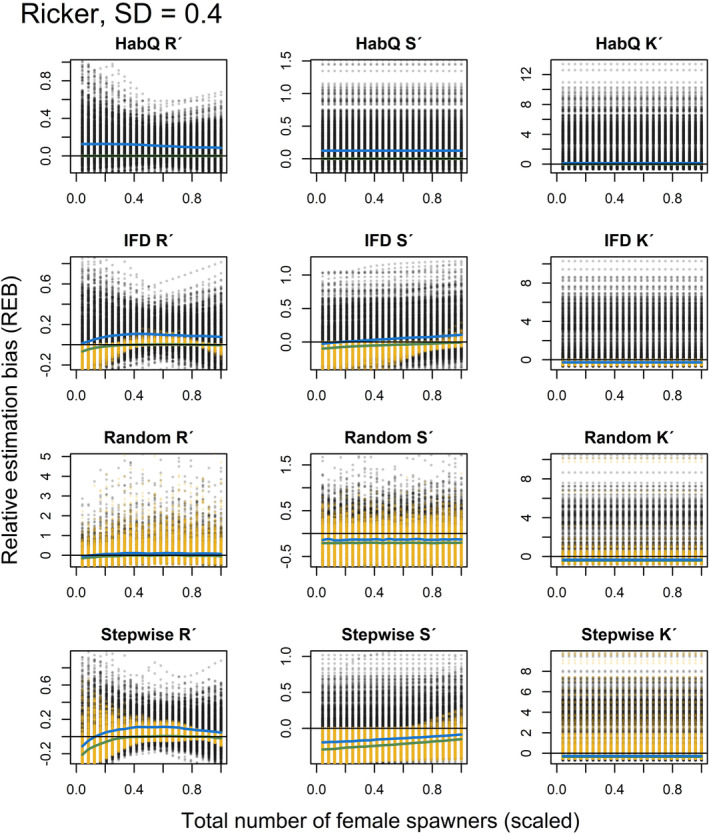
REB for *R*′, *S*′ and *K*′, based on low spawner abundance (*K*_*tot*,*j*_/2) without (yellow dots) and with added measurement error (SD = 0.4, black dots), for a Ricker SR relationship. Green and blue solid lines show the median REB without and with measurement error, respectively. Black solid line shows zero REB. Note the different scales on the Y‐axis. For clearer visualization, female abundance (x‐axis) is displayed as a proportion of the evaluated maximum abundance.

**FIGURE 10:**
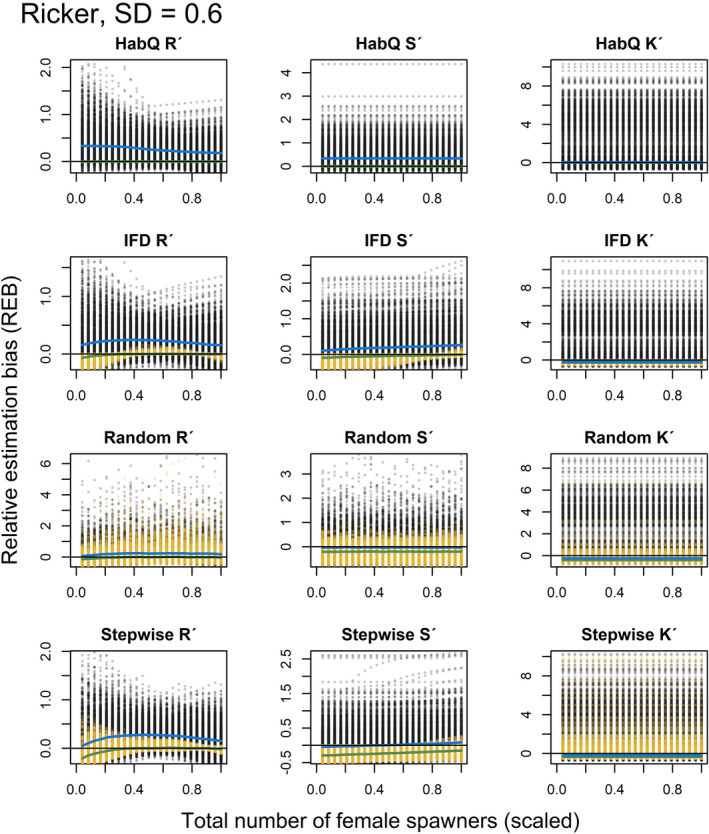
REB for *R*′, *S*′ and *K*′, based on low spawner abundance (*K*_*tot*,*j*_/2) without (yellow dots) and with added measurement error (SD = 0.6, black dots), for a Ricker SR relationship. Green and blue solid lines show the median REB without and with measurement error, respectively. Black solid line shows zero REB. Note the different scales on the Y‐axis. For clearer visualization, female abundance (x‐axis) is displayed as a proportion of the evaluated maximum abundance.

For the analysis of REB at lower population abundances and different levels of measurement error, the results are presented both graphically (Figures 5, 6, 7, 8, 9, 10) and as numbers (Table 2). The figures presented in the main manuscript compare REB introduced by zero measurement errors (i.e. only spawning behavior), and REB introduced by measurement errors with SD = 0.2, 0.4 and 0.6 (i.e. spawning behavior and added measurement error) for the Beverton‐Holt (Figures 5, 6, 7) and Ricker SR functions (Figures 8, 9, 10).

Even if additional measurement error affected the magnitude of the REB, the different spawner behaviors influenced the REB differently, and for some of the spawner behaviors, the spawning behavior explained most of the REB (Figures 5, 6, 7, 8, 9, 10, Table 2). What was most interesting was that the average REB for the *R*_*j*_′ was more or less equal for the four different spawner behaviors and SR functions, whereas the average REB in *K*_*j*_′ and *S*_*j*_′ was higher for stepwise and random spawner behaviors (Table 2). Moreover, the average REB in *K*_*j*_′ and *S*_*j*_′ estimates was increased with increasing levels of measurement error for HabQ and IFD spawner behaviors, whereas the average REB decreased with increasing levels of measurement error for the stepwise and random spawner behaviors (Table 2).

### 3.7 HabQ

For a HabQ spawner behavior, measurement error with SD = 0.2, 0.4 and 0.6 explained the majority of the REB for the estimated *R*_*j*_′, *S*_*j*_′ and *K*_*j*_′ (Table 2). This was consistent for both the Beverton‐Holt and the Ricker SR relationship (Figures 5, 6, 7, 8, 9, 10). In Figures 5, 6, 7, 8, 9, 10, this is visualized by the majority of the visible points being black. This means that for stocks with a HabQ type of spawner behavior, data containing low spawner abundance, and measurement error (SD ≥0.2); the measurement errors will generate larger REB than the underlying spawner behavior.

### 3.8 Ideal free distribution

For an IFD spawner behavior, all three levels of measurement error resulted in a large variation in the REB of *R*_*j*_′, *S*_*j*_′ and *K*_*j*_′ (Figures 5, 6, 7, 8, 9, 10, Table 2). Both the median REB and the variation in REB were larger than what was produced by the underlying spawner behavior (zero measurement error) (Figures 5, 6, 7, 8, 9, 10, Table 2). These results were consistent for both SR relationships. In estimates of *R*_*j*_′ and *S*_*j*_′ the REB was influenced by the spawner abundance (Figures 5, 6, 7, 8, 9, 10), where larger REB was introduced at low spawner abundance.

### 3.9 Random

For a random spawning behavior and a moderate measurement error (SD = 0.2), most of the REB in *R*_*j*_′, *S*_*j*_′ and *K*_*j*_′ were explained by the spawner behavior. This was consistent for both SR relationships (Figures 5, 8). However, increased measurement error (SD > 0.2) generated larger and more variable median REB (Figures 6, 7, 9, 10, Table 2). For all levels of measurement error, the REB was constant over the spawner abundance range, which means that the REB was not dependent on the abundance level. Compared with the IFD and HabQ the random spawner behavior introduced a larger median REB in estimates of *S*_*j*_′ (Figures 5, 6, 7, 8, 9, 10).

### 3.10 Stepwise

For a stepwise spawner behavior and a majority of the REB in estimated *R*_*j*_′ was explained by the spawner behavior compared with measurement error (SD = 0.2, 0.4 and 0.6, Figures 5, 6, 7, 8, 9, 10, Table 2), except for a Ricker SR relationship with SD = 0.6 (Figure 10, Table 2). The estimates of *S*_*j*_′ was also largely influenced by the spawner behavior at low measurement error (SD = 0.2), whereas larger measurement error (SD = 0.4 and 0.6) generated high variation in REB (Figures 5, 6, 7, 8, 9, 10, Table 2). Even if the median of the REB in the estimated *S*_*j*_′ was almost equal between the different measurement error levels (SD = 0, 0.2, 0.4 and 0.6), REB without measurement error (SD = 0) introduced REB that was almost exclusively negative, whereas the other three levels of measurement error introduced both negative and positive REB (Figures 5, 6, 7, 8, Table 2). Estimates of *K*_*j*_′ introduced large variations in REB, independently of the SR relationship and spawner abundance level (Figures 5, 6, 7, 8, 9, 10, Table 2).

## 4 DISCUSSION

Two of the spawner behaviors evaluated (HabQ and IFD) introduced no or negligible bias in the estimates of total recruitment, survival rate and maximum potential recruitment, whereas the other two spawner behaviors (random and stepwise) generated considerable REB. These results were consistent for both the Beverton‐Holt and Ricker SR relationships. These general results were also consistent with and without additional bias (i.e. measurement error). Our results, therefore, suggest that for some underlying spawner behaviors the SR relationship might not be well approximated even when local recruitment is based on one of these two functions. Depending on the habitat selection pattern, this miss‐specification might ultimately yield biased parameter estimates for two extensively used SR functions (i.e. Beverton‐Holt and Ricker).

For the two common SR functions studied herein, spawning individuals in the population are typically assumed to distribute homogeneously across all potential spawning sites in a system, with all offspring suffering the same average mortality independent of where they were born. These assumptions are probably not realistic for most fish species, and there are studies suggesting alternative spawner behaviors for at least some species (Bouchard et al., 2018; Falcy, 2015; Finstad et al., 2013; Haugen et al., 2006; Huntsman et al., 2017; MacCall et al., 2019). The four distribution patterns evaluated in this study are perhaps rough simplifications of the real world, but our results clearly demonstrate that depending on the spawner behavior in combination with environmental conditions in terms of density‐independent survival rates and carrying capacities, estimates of population productivity can be biased using these two common SR functions´.

When the spawner behavior was proportional to the local carrying capacities (i.e. HabQ distribution), parameters were estimated with high accuracy and precision over the entire spawner abundance range (i.e. low REB). Under a more realistic management scenario using a lower spawner abundance range and additional measurement error the median estimates of *R´*, *S´* and *K´* did not change remarkably, only variation in REB, which could be explained by the level of measurement error that was added. This indicates that under a HabQ spawner behavior, the Beverton‐Holt and Ricker SR functions are consistent with the assumption that reproducing individuals distribute in a spawning system according to local carrying capacities, where abundance occupation of all spawning sites is expected even at low spawner abundance (see derivation in Appendix S1). This type of spawner behavior has been observed in chinook salmon (*Oncorhynchus tshawytscha*) where habitat quality can override a strict homing spawner behavior (Cram et al., 2013). However, this relationship no longer holds when S0 differs between habitats. Since S0, among other things includes losses due to predation, one might suspect that S0 differs between spawning areas, and our conclusion of negligible bias in the global SR function for the HabQ behavior might therefore be over‐optimistic.

The Ideal free distribution has been suggested to hold as the spatial distribution for many marine fish species (Shepherd & Litvak, 2004). Our results show that an IFD spawner behavior generated relatively low REB on average, but with a larger variation compared with the HabQ. It is important to note that each dot in Figures 3, 4, 5, 6, 7, 8, 9, 10 represents recruitment from one spawner abundance level in one environment. So a larger variation in REB illustrates that for some environments (habitat parameter settings) we might risk considerable bias if we do not consider the underlying spawner behavior in the SR functions. Moreover, the REB in *R′* and *S′* varied over the spawner abundance range, which means that the magnitude of REB was influenced by the spawner abundance level, where at low spawner abundance *R′* and *S′* were underestimated, but at high spawner abundance levels the REB was almost zero. These results were consistent also when only low spawner abundance range data was used and measurement errors were added. Therefore, for populations at low abundance levels also an IFD might introduce bias in estimates of SR relationships if the spawner behavior is ignored.

The variation and magnitude of REB were more pronounced in the random spawner behavior which is perhaps not surprising since the density in each spawning site was randomly assigned, meaning that some sites were underutilized, whereas others were over‐utilized over the full spawner abundance range. This was also reflected in the low spawner abundance data analysis with additional measurement errors added where a large proportion of the variation in REB could be explained by the random behavior, even at the highest levels of measurement errors added (SD = 0.6). Maunder and Deriso (2013) suggested a spatial extent SR function resembling a Beverton‐Holt SR relationship that assumed a random distribution of spawners. However, as also Maunder and Deriso (2013) discuss, a random spawner behavior might perhaps not be realistic.

A stepwise spawner distribution is perhaps not a realistic spawner distribution on its own either, but stepwise dispersal in fish has been observed in empirical studies; e.g. reflecting spillover processes (Abesamis & Russ, 2005), spawner dispersal (Finstad et al., 2013) and density‐dependent habitat expansion (Bartolino et al., 2011). The stepwise spawner behavior generated a large median REB with considerable variation. This was consistent for all analyses and SR functions. Moreover, the stepwise spawner behavior explained the majority of the variation in REB even when additional measurement error was introduced. These results indicate that in some environments a stepwise spawner behavior may generate estimation bias in vital SR relationship estimates, which should be of concern for stock assessments. A Larger REB was generated in estimates of the maximum survival rate parameter (*S´*), compared with the total carrying capacity (*K*′). This was consistent using both the full and low spawner abundance range, with and without measurement error added, and for both SR relationships. The high accuracy and precision in the estimates of *K*′ is probably due to that the spawner abundance range was sufficient to inform the SR functions of the peak (Ricker) or asymptote (Beverton‐Holt) part of the SR relationship.

Even if *K*′ is essential in SR functions, most exploited fish stocks are probably far from the true carrying capacity of the system, which makes estimates of the *S*′ parameter arguably more important for population dynamics modeling and stock assessment (Myers, 2001). The *S*′ parameter is the initial slope of the SR curve and can be interpreted as the maximum reproductive rate of a stock, which makes it an important parameter in fisheries assessment and management (Myers, 2001). Our results show that the population‐level survival rate is not only affected by spawner behaviors and the SR relationship (Beverton‐Holt or Ricker), but also by spawner abundances for a given distribution pattern. As an example, IFD, random and stepwise spawner behaviors generated underestimates of the survival rates at low spawner abundances but negligible REB at high spawner abundance levels. Thus, even if we would have data covering the whole range of population density, we still risk underestimating the survival rate when population abundance declines. Interestingly, the median REB in the survival rate estimates were reduced for a random and stepwise spawner behavior when additional measurement errors were added. The majority of the REB in survival rate was mainly negative and additional measurement error introduced positive REB, which generated a median REB closer to the predefined parameter values. Therefore, measurement errors might in these cases make estimates less biased. Furthermore, for fish species that have both spatially constrained and unconstrained life stages, climate change might induce a further separation in stage‐specific habitat usage (Ciannelli et al., 2022). This could lead to a reduction in the habitat range where local spawning sites might be inaccessible or unused in the future.

### 4.1 Management perspectives

Even when factors contributing to lifetime reproductive output (e.g. fecundity, maternal age and size structure) are accounted for in SR functions, estimates of recruitment often remain highly variable (Green, 2008). Our results suggest that the distribution of spawners might influence such variability. However, our analysis with additional measurement error, and with SR data covering only low spawner abundance levels, shows that for a HabQ and IFD spawner behaviors, the resulting increase in bias, might mask the effects of the underlying spawner behavior (e.g. generate higher variation in REB than the spawner behavior). Most stock‐recruit data sets for managed fish stocks lack contrast in spawner abundances, and rather consist of relatively short time series at low or intermediate spawner abundances (Hilborn & Walters, 2015). Moreover, additional sources of bias, e.g. environmental variation, time‐series bias, shifting productivity regimes, and observation and (or) process bias are common in SR data (Haddon, 2001; Maunder & Piner, 2015; Quinn & Deriso, 1999; Vert‐pre et al., 2013; Walters, 1985). For stocks lacking contrasting data and with measurement errors (SD > 0.2) the bias caused by the underlying spawner behavior might therefor be of subordinate concern compared with that related to the measurement errors if the spawner behavior is either HabQ or IFD. In contrast, for both the Beverton‐Holt and Ricker functions, a stepwise or random spawner behavior may introduce additional uncertainties that are not explained by the measurement error (i.e. variation in the REB is higher than what is added by the measurement error) Measurement errors are most often accounted for in stock assessment (Brooks et al., 2019), but additional estimation bias might be introduced by the underlying spawner behavior. The majority of the REB in our analyses was negative, which means that the productivity was underestimated. Underestimation of population productivity may lead to underutilization of populations, whereas overestimation may tend to produce overly‐optimistic management advice (Conn et al., 2010; Hilborn et al., 2015), ultimately also resulting in lost yield. The extent of the loss can depend on the magnitude of the bias. Our results show that the magnitude and direction (under−/overestimation) of the parameter estimation bias depends on the spawner behavior and varies over the population abundance range. This is mainly an effect of the under−/overutilization of local spawning sites depending on the spawner distribution pattern. Irregular productivity regimes, not only depending on abundance, have been raised as an important factor in fisheries management (Vert‐pre et al., 2013). Our results suggest that the spawner behavior might be one component contributing to this irregularity in productivity and that the productivity can vary according to population density.

For habitat restoration, ignoring the underlying distribution patterns of spawners may directly affect the outcome of restoration objectives, since habitats could be over‐ or underutilized compared with the common SR assumption, where spawners would utilize all potential spawning sites instantly and distribute homogenously across all spawning sites. This could ultimately make predictions of a system's productivity biased. Notably, if spawners follow a stepwise pattern and distribute according to the recruitment migration distance and the local carrying capacity, colonization of new habitats would probably be delayed compared with a more homogeneous spawner distribution (Einum et al., 2008b; Huxel & Hastings, 1999). Moreover, our results show that productivity is not stationary over the spawner abundance range. Results based on global SR functions may thus deviate considerably from the actual SR relationship of the global population in certain population ranges, where under−/overestimations of the survival parameters depend on the distribution pattern. Therefore, for recovering exploited populations, knowledge of the underlying distribution pattern of reproducing individuals could be important for accurate stock‐recruit parameter estimation and robust predictions of population development.

### 4.2 Directions for future research

Spawner behaviors can be modeled and included in stock assessments; however, to do so, the specific behavior needs to be known, since the variability depends on the population (stock) specific spawner behavior. Empirical studies show that different fish species may display different distribution patterns (e.g. Foldvik et al., 2010; Huntsman et al., 2017; Langangen & Stige, 2021), indicating the lack of a general spawner distribution model that can be applied in stock assessments. It is beyond the scope of this study to provide a general suggestion on how to statistically handle spawner behavior patterns in stock assessments, but more stock‐specific knowledge of spawner distribution appears warranted, which can be obtained for some species via e.g. telemetry studies (Dean et al., 2014) or nest counts (Finstad et al., 2013). As an extension to our present study, we are currently exploring if the spawner distribution behavior of Atlantic salmon could be detected using empirical spatio‐temporal data on juvenile abundance (from electrofishing) together with spawner counts. This could be one way forward to gain more knowledge of the distribution behavior before additional data from other methods are available.

### 4.3 Main conclusion

Using theoretically possible spawner distribution patterns, our results show that, when applying commonly used SR functions, the underlying distribution patterns of spawning individuals can affect estimates of SR parameters that govern the productivity of exploited fish stocks. Moreover, for stock assessments, the results from this simulation study suggest that any underlying distribution pattern that deviates from a homogenous distribution of spawning individuals can introduce systematic bias in parameter estimates, where the magnitude of estimation bias depends on the underlying spawning distribution pattern. For some of the spawner behaviors evaluated, realistic levels of measurement error (SD ≥ 0.2) would introduce larger bias than resulting from the spawner behavior, which makes measurement errors of higher concern from a management perspective. Moreover, the majority of the estimation bias was of a precautionary nature (underestimation), which means that violating the spawner distribution assumption will not lead to unsustainable harvest rates but rather a potential loss of yield. To reduce potential estimation bias, further research into stock‐specific spawner distribution patterns is needed.

## AUTHOR CONTRIBUTIONS

**Stefan Skoglund:** Formal analysis (lead); methodology (equal); validation (lead); visualization (lead); writing – original draft (lead); writing – review and editing (lead). **Rebecca Whitlock:** Conceptualization (equal); formal analysis (equal); methodology (equal); supervision (equal); validation (equal); visualization (equal); writing – review and editing (equal). **Erik Petersson:** Supervision (equal); writing – review and editing (equal). **Stefan Palm:** Conceptualization (equal); supervision (equal); validation (equal); writing – review and editing (equal). **Kjell Leonardsson:** Conceptualization (equal); methodology (equal); supervision (equal); validation (equal); visualization (equal); writing – review and editing (equal).

## FUNDING INFORMATION

The project is funded by the Swedish University of Agricultural Science, without any funding ID‐number.

## Supporting information


Appendix S1:

## Data Availability

The data that support the findings of this study are openly available in figshare at https://figshare.com/account/home#/projects/154619, reference number 154619.
