# Supplementary material for: From spawner habitat selection to stock‐recruitment: Implications for assessment
Source: Ecol Evol. 2022 Dec 28;12(12):e9679. doi: 10.1002/ece3.9679 (PMC9797469; doi:10.1002/ece3.9679)
Supplement: Supplementary file 1 — Appendix S1: [file ECE3-12-e9679-s001.docx]

**Supplementary Information (SI)**

Here is the derivations proving that the Beverton-Holt SR function (valid also for Ricker)

s = density independent survival during the juvenile phase, e.g. in the river. Here assumed to be the same for all spawning grounds.

$N=\sum_{i=1}^{\max} n_{i}$, (spawners and their fecundity)

$K=\sum_{i=1}^{\max} k_{i}$, (carrying capacities)

The proof below is for the Beverton-Holt SR function, but the same method can be used to verify that it holds also for the Ricker SR function.

Show that the left-hand side (LHS) equals the right-hand side (RHS) of the two alternative formulations of Beverton-Holt functions below, which both aggregate spawners (n_i_) from an arbitrary number (max) of spawning grounds each with its unique carrying capacities k_i, i=1-max_. The LHS version sums the BH functions (local recruitment) from each local spawning ground, while the RHS version is a single traditional BH function that directly calculates the total recruitment by using the total number of spawners as well as the total carrying capacity.

$\sum_{i=1}^{\max} (s*{n_{i}}/{(1+s*{n_{i}}/{k_{i}})})=s*\sum_{i=1}^{\max} n_{i}/(1+s*\sum_{i=1}^{\max} {n_{i}}/{\sum_{i=1}^{\max} k_{i}})$

Substitute $\sum_{i=1}^{\max} n_{i}$ with N, and $\sum_{i=1}^{\max} k_{i}$ with K into the RHS

$\sum_{i=1}^{\max} (s*{n_{i}}/{(1+s*{n_{i}}/{k_{i}})})=s*N/(1+s*N/K)$

Assume that the spawners select spawning grounds directly proportional to the carrying capacities of the spawning grounds (habQ scenario).

This means that for example n_i_=N*k_i_/K spawners will select spawning ground i.

Replace n_i_ with N*k_i_/K in the LHS.

$\sum_{i=1}^{\max} (s N{{k_{i}}/K}/{(1+s*N{{k_{i}}/K}/{k_{i}})})=s*N/{(1+s*N/K)}$

Simplify

$s*N/{K*}\left( \sum_{i=1}^{\max} k_{i} \right)/(1+s*N/K)=s*N/{(1+s*N/K)}$

Replace $\sum_{i=1}^{\max} k_{i}$ with K

$s*N/K*K/{(1+s*N/K)}=s*N/{(1+s*N/K)}$

Simplifying after the last substitution gives LHS=RHS

$s*N/{(1+s*N/K)}=s*N/{(1+s*N/K)}$

Conclusion

The two alternative formulations of the Beverton-Holt function are identical given the assumptions made. Following the description above it turns out that it is valid also for the Ricker function. Hence, the traditional Beverton-Holt as well as the Ricker stock recruitment functions allow for heterogenous recruitment areas (varying size or quality) when the spawners choose the spawning grounds in proportion to the size or quality of the available spawning grounds and when the density independent survival of the offspring is the same in each area. In fact, there is some possibility for varying density independent offspring survival, but that requires s0_i_*s1_i_=s0_j_*s1_j_ for all i and j. Moreover, as the total spawner density increase the influence of the density independent survival parameters decreases, implying that the LHS and the RHS of the above expression will be fairly equal even when there are some deviation in density independent survival between the areas, as can be seen in the habQ graphs showing a small bias of the global Beverton-Holt and Ricker SR-functions at increasing female numbers.
